# Depression among survivors of 1988 Spitak earthquake in Armenia: a prospective cohort study

**DOI:** 10.1093/eurpub/ckac130.179

**Published:** 2022-10-25

**Authors:** A Demirchyan

**Affiliations:** Gerald and Patricia Turpanjian College of Health Sciences, Yerevan, Armenia

## Abstract

**Background:**

Mental health-related consequences of disasters can be very long lasting. Depression is one of the most prevalent psychopathologies among disaster survivors. Yet, compared to PTSD, it is studied much less among disaster victims. Studies of depression decades after the exposure are extremely rare. This study evaluated the prevalence and predictors of depression in a prospective cohort of Spitak earthquake survivors 23 years after the event.

**Methods:**

A geographically stratified urban sub-sample of 1785 individuals underwent all three baseline waves of the cohort study during 1990-1992 and provided detailed sociodemographic, earthquake exposure and physical and mental health-related information. In 2012, 83.3% (n = 1487) of this subsample was traced and 40.6% (n = 725) interviewed. Depression status was measured via validated and adapted Armenian-language CES-D scale. A fitted linear regression model identified predictors of depression score in 2012.

**Results:**

Twenty-three years after the exposure, the rate of depression in this cohort was 25.4%. Depression was highly comorbid with anxiety (62.0%) and PTSD (36.8%). Factors positively associated with depression score included number of stressful life events (95% CI 0.33, 1.20), poor self-rated health (95% CI 2.45, 5.40), earthquake-related deaths in the family (95% CI 0.12, 3.80), and strong phobia and fear at baseline (95% CI 0.51, 2.96). Age (95% CI -0.15, -0.06), social support score (95% CI -0.83, -0.55), quality of life score (95% CI -15.83, -10.77), and being married (95% CI -2.61, -0.35) were protective for depression. The fitted model explained 46.6% of the variance in the depression score.

**Conclusions:**

This study found an increased prevalence of depression among earthquake survivors over two decades after the exposure. The identified potentially modifiable predictors of long-term depression create prerequisites for planning better targeted mental health recovery interventions among disaster survivors.

**Key messages:**

- Traumatic deaths in the core family, strong emotional response to the event, and lack of social support predicted depression over two decades after the disaster.
- A considerable proportion of survivors reported long-term depressive symptoms highly comorbid with anxiety and PTSD, underscoring the need for screening of all three traits and intervening early-on.

